# Coordinated epigenetic regulation of *Engrailed-1* by the chromatin remodelers *Smarca1* and *Smarca5* mediates cerebellar morphogenesis

**DOI:** 10.1186/1756-8935-6-S1-P105

**Published:** 2013-04-08

**Authors:** Matias Alvarez-Saavedra, Pamela Lagali, Keqin Yan, Emile Hashem, Alan Mears, Yves De Repentigny, Valerie A Wallace, Rashmi Kothary, Tomas Stopka, Arthur I Skoultchi, David J Picketts

**Affiliations:** 1 Regenerative Medicine Program, Ottawa Hospital Research Institute, Ottawa, ON, Canada, KlH8L6; 2 Vision Program, Ottawa Hospital Research Institute, Ottawa, ON, Canada, K1H 8L6; 3 Department of Cellular and Molecular Medicine, University of Ottawa, ON, Canada; 4 Department of Biochemistry, Microbiology & Immunology, University of Ottawa, ON, Canada; 5 Institute of Pathologic Physiology, First Faculty of Medicine, Charles University in Prague, Prague, Czech Republic; 6 Department of Cell Biology, Albert Einstein College of Medicine, Bronx, NY, USA

## Background

Morphological patterning of the cerebellum requires precise changes in *Engrailed* homeotic gene expression yet the mechanisms controlling this process remain elusive. Here, we show that the *Iswi* chromatin remodeling proteins, *Smarca5* and *Smarca1*, are required for the dynamic regulation of *Engrailed-1* (*En1*). Conditional *Smarca5*-null mice display abnormal cerebellar foliation, ataxia-like symptoms and young mortality. Postnatal granule neuron progenitor expansion and Purkinje cell (PC) development are compromised and attributed to loss of *En1* expression. Mutants survive to early adulthood via upregulation of *Smarca1* and restoration of *En1* expression in PCs, while ablation of both *Iswi* genes results in lethality at birth. During late cerebellar development, we observe co-binding of the *Iswi* proteins at the *En1* locus and an altered H2AZ/H3.3 chromatin profile that accompanies changes in *En1* expression.

## Materials and methods

Through mouse ES cell homologous recombination, we targeted exon 5 of the *Smarca5* gene that encodes for part of the helicase domain, thereby allowing us to conditionally ablate Smarca5 expression in the central nervous system (CNS). We ablated Smarca5 expression in CNS progenitors using the Nestin-Cre driver, as well as in Purkinje neurons of the cerebellum using the PCP2-Cre driver mouse line. We also ablated expression of both mammalian Iswi proteins by breeding to a *Smarca1*-null mouse that we have previously characterized [1].

Conclusions Our results support an epigenetic mechanism in which *Iswi*-mediated histone variant exchange modulates *En1* expression levels to subsequently control cerebellar morphogenesis.
